# A Dominant Negative ERβ Splice Variant Determines the Effectiveness of Early or Late Estrogen Therapy after Ovariectomy in Rats

**DOI:** 10.1371/journal.pone.0033493

**Published:** 2012-03-13

**Authors:** Jun Ming Wang, Xu Hou, Samuel Adeosun, Rosanne Hill, Sherry Henry, Ian Paul, Ronald W. Irwin, Xiao-Ming Ou, Steven Bigler, Craig Stockmeier, Roberta Diaz Brinton, Elise Gomez-Sanchez

**Affiliations:** 1 Department of Pathology, University of Mississippi Medical Center, Jackson, Mississippi, United States of America; 2 Department of Psychiatry and Human Behavior, University of Mississippi Medical Center, Jackson, Mississippi, United States of America; 3 Program in Neuroscience, University of Mississippi Medical Center, Jackson, Mississippi, United States of America; 4 Department of Pharmacology and Toxicology, University of Mississippi Medical Center, Jackson, Mississippi, United States of America; 5 GV (Sonny) Montgomery VA Medical Center, University of Mississippi Medical Center, Jackson, Mississippi, United States of America; 6 Department of Medicine, University of Mississippi Medical Center, Jackson, Mississippi, United States of America; 7 Department of Pharmacology and Pharmaceutical Sciences, University of Southern California, Los Angeles, California, United States of America; Oklahoma Medical Research Foundation, United States of America

## Abstract

The molecular mechanisms for the discrepancy in outcome of initiating estrogen therapy (ET) around peri-menopause or several years after menopause in women are unknown. We hypothesize that the level of expression of a dominant negative estrogen receptor (ER) β variant, ERβ2, may be a key factor determining the effectiveness of ET in post-menopausal women. We tested this hypothesis in ovariectomized nine month-old (an age when irregular estrous cycles occur) female Sprague Dawley rats. Estradiol treatment was initiated either 6 days (Early ET, analogous to 4 months post-menopause in humans), or 180 days (Late ET, analogous to 11 years post-menopause in humans) after ovariectomy. Although ERβ2 expression increased in all OVX rats, neurogenic and neuroprotective responses to estradiol differed in Early and Late ET. Early ET reduced ERβ2 expression in both hippocampus and white blood cells, increased the hippocampal cell proliferation as assessed by Ki-67 expression, and improved mobility in the forced swim test. Late ET resulted in either no or modest effects on these parameters. There was a close correlation between the degree of ERβ2 expression and the preservation of neural effects by ET after OVX in rats, supporting the hypothesis that persistent elevated levels of ERβ2 are a molecular basis for the diminished effectiveness of ET in late post-menopausal women. The correlation between the expression of ERβ2 in circulating white blood cells and brain cells suggests that ERβ2 expression in peripheral blood cells may be an easily accessible marker to predict the effective window for ET in the brain.

## Introduction

Most women in developed countries now live 25 to 30 years, one third of their lives, after menopause, during which time they are at increased risk for cardiovascular disease, osteoporosis and dementia. Research into the etiology of menopausal dementia leading to the development of effective prevention and therapeutic strategies, will thereby improve women's overall health and quality of life, and significantly reduce the public healthcare burden.

Basic science studies and observational trials [1]–[6] suggested that estrogen therapy (ET) would prevent cognitive decline associated with menopause, however, this was challenged by the results from the Women's Health Initiative and the ancillary Memory Study (WHIMS) in 2002 [7], [8]. Among reasons cited for the discrepancy were differences in the type of estrogenic compounds used, routes of administration, cyclic versus continuous regimens, concomitant use of progestins, and the time after menopause that the ET was initiated. The latter led to the hypothesis of a critical period, or therapeutic window, during which ET must be initiated to obtain maximum benefit [1], [5], [9], [10]. To date, the molecular basis of this hypothesis and the boundaries of the clinical therapeutic window are unknown.

Many of the effects of estrogens are mediated by estrogen receptors (ER), primarily ERα and ERβ, which are encoded by different genes. Both ERα and ERβ are expressed in the brain. ERβ is thought to be more important in sleep disturbance, irritability, depression and anxiety, panic disorders, and cognitive dysfunction [11]–[16]. The ERβ transcripts are spliced into different mRNAs and translated into ERβ variants with different biological functions depending physiological or pathological conditions. One of these, hERβ2/hERβcx found in humans and non-human primates, contains unique 26 amino acid (aa) residues which replace the aa residues encoded from exon 8 in the C-terminal part of the ligand binding domain. hERβ2 forms dimers with other ER, but is unable to bind ligands or coactivators and has no transcriptional activity in reporter assays[17], [18]. Ectopic expression of hERβ2 inhibits ERα-mediated gene transcription and cell proliferation *in vitro* in MCF cells [19]. Hence it is considered a dominant negative isoform. Swaab's group has demonstrated the expression of the alternatively spliced ERα mRNAs in post-mortem brain of Alzheimer's Disease and that the dominant negative deletion (del.) 7 isoform appeared to be the major splice variant [20].

Several ERβ splice variants have been identified in the rat hippocampus. One of these, rERβ2, has an additional 54 bp nucleotides coding for extra 18 amino acids within the ligand binding domain that changes the conformation and reduces the binding affinity of rERβ2 to estradiol by up to 30-fold [21], [22]. In addition, rERβ2 also exhibits weaker interactions with TIF2 and RAP250, two transcription coactivators [21], [22]. The low binding affinity to estradiol and the ability to interact with coactivators make rERβ2 a dominant negative receptor in the rat [23], [24]. Therefore, although the rERβ2 and hERβ2 do not share the same spliced aa residues, both differ from ERβ in the C-terminal ligand binding domain, both result in diminished ligand binding and both preferentially dimerize with ERα, decreasing normal ER-mediated functions [19].

It has been reported that ERβ2 levels increase in the hippocampus [25], [26], and pituitary [27] of OVX rats. This shift to prominent expression of the dominant negative receptor is reversible while there are still enough functional ERβ, but at some yet to be defined point, the level of ERβ2 expression disrupts normal ERβ activity. We hypothesize that ERβ2 expression increases with the duration of gonadal hormone deprivation and that the point at which ERβ2 levels disrupt ER-mediated functions is the molecular mechanism underlying the closing of the therapeutic window for estrogen therapy. To test this hypothesis, rats were ovariectomized at 9 months of age and studied after Early ET (E_2_ treatment starting 6 days after OVX, equivalent to human early post-menopause [28], [29]) and Late ET (E_2_ treatment starting 180 days after OVX, equivalent to starting 10–20 years after menopause, as was done in the WHIMS trial). ERβ2 levels in peripheral blood cells were compared to those in the brain to determine whether these could be a valid marker for ERβ2 expression in the brain.

## Materials and Methods

### Animal and treatments

The animal protocol (#1168) was approved by the University of Mississippi Medical Center Animal Care and Use Committee and conformed to National Institutes of Health guidelines. UMMC is an AAALAC accredited facility. Six month old female or retired breeder (9 month-old) Sprague-Dawley rats were purchased from Charles River (Harlan Laboratories, Indianapolis, IN) and were housed and maintained on a 12/12-hour light/dark cycle (lights on 7:00 AM) and provided with unlimited access to food and water in the Laboratory Animal Facilities at the University Mississippi Medical Center.

### Ovariectomy

The rats were anaesthetized with ketamine (100 mg/kg) and xylazine (10 mg/kg). The incision sites were shaved and cleansed with povidone iodine solution. Standard aseptic procedures were observed. Dorsal incisions were made in the lumbar region to reveal the dorsal fat pads covering the ovaries. Ovaries were removed after compression of the ovarian pedicle. After ovariectomy, the incisions were sutured. Rats were given Carprofen (subcutaneous, 5 mg/kg, once/day for 2 days), and returned to home cages for the number of days required by the experiment at which time they received E_2_ or vehicle injections.

### Groups

**Figure 1:**
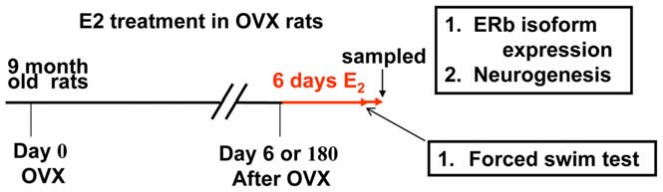
Experimental flow chart for determining the impact of Early and Late estrogen therapy. Nine-month-old Sprague Dawley rats were ovariectomized and estradiol treatment was initiated 6 days (Early ET) or 180 days (Late ET) after OVX. After 6 days of treatment including two days behavioral tests, the rats were humanely sacrificed and brain and blood samples collected.

The effects of removing ovarian hormones on the expression of ERβ and ERβ2 were first evaluated in adult reproductive (6 month-old) naïve, sham OVX and OVX rats 14 days after surgery. Rats begin to show irregular estrous cycles at 9–12 months, as defined by prolonged cycles, interspersed with the normal 4–5 days rhythm [28], [29]. As aging occurs, these animals transition into an acyclic, persistent anestrus status, in which ovulation has ceased [28], [29] at 15 to 23 months of age. Therefore, in order to further study the role of ERβ2 in Early and Late ET in post-menopausal females, female Sprague-Dawley rats, at 9 months old (compatible to human perimenopause), were ovariectomized (OVX, a rodent menopausal model) or sham-OVX. Estradiol treatments were initiated on day 7 (Early ET) or day 181 (Late ET) after OVX (Figure 1). In both Early and Late ET cohorts, 8 rats were sham-OVX, and 17 were OVX. Of the 17 OVX rats, 9 were treated with estradiol, while the other 8 were received vehicle.

### Treatment

Rats were subcutaneously injected with estradiol, 30 µg/kg body weight or vehicle for 5 days (once per day). Although it has been reported by Tanapat that 10 µg/kg, but not 50 µg/kg, was the best concentration for promoting the proliferation of neural progenitor cells in the SGZ in OVX female rat hippocampus [30], the effects of estradiol at the doses between 10 and 50 µg/kg were not reported. In our previous dose response studies, we found that the concentration of 30 µg/kg estradiol showed the highest effects on neuroprotection and neurogenesis [16], [31]. One day after the last estradiol injection, rats were assigned for a forced swim test (5 minutes) in random order. Animals were humanely sacrificed 7 days after the first estradiol injection and leukocytes, plasma, uterus, and brain were collected. One hemisphere of the brain was immediately dissected and several cortical regions and the hippocampus were frozen in liquid nitrogen and stored at −80°C for mRNA and protein assay. The other hemisphere was fixed in 4% paraformaldehyde (PFA).

### FACS assay for Ki-67 positive cells

The assay was performed as previously described [32]. Briefly, the hippocampus was extracted from the PFA fixed brain hemispheres using consistent anatomical landmarks as criteria for dissection as described by Bilsland [33]. The white membranes were removed to avoid the contribution from subventricular zone and rostral migratory stream proliferative pools. Nuclei were extracted from the homogenate from individual hippocampus. Nuclei aliquots were resuspended in 200 µL of a 0.2 M solution of boric acid, pH 9.0, and heated for 1 h at 75°C for epitope retrieval. Subsequently, nuclei were incubated for 24 h at 4°C in primary antibodies (1∶500 for polyclonal anti-Ki-67, abcam, ab15580) and in CY2-conjugated goat anti-rabbit IgG secondary antibody (1∶100 in PBS; Jackson Immuno Reasearch Labs, Inc.) for 2 h. An aliquot of the nuclear suspensions (2.5 ul) was stained with propidium iodide (PI), a fluorescent molecule that stoichiometrically binds to DNA by intercalating between the bases with no sequence preference, and checked under fluorescent microscope to verify the immunolabeling quality. The remaining cell suspension was diluted to 500 µL for flow cytometry assay using a Beckman FC 500 System with CXP Software. To avoid counting bias, we registered the presence or absence of Ki-67 immunoreactivity for all of the PI-stained nuclei samples until 10,000 PI-stained nuclei were examined. Nuclear density was estimated by counting the PI positive particles.

### Extraction of leukocytes protein from whole blood

To translate the concept that ERβ2 expression predicts the effectiveness of ET into a clinically useful guideline, we measured ERβ2 expression in circulating white blood cells (WBC). Ammonium chloride solution (0.17 M at room temperature) was added to each blood sample in a volume 1∶9 to lyse red blood cells and the samples incubated for 5 minutes in a rotator. The red blood cells would not be completely lysed if the solution was cold and the white blood cells began to lyse if the incubation exceeded 5 minutes. The suspension of WBC and lysed RBCs was centrifuged for 5 minutes at 2000 RPM. The supernatant was discarded and the pellet washed in ice old PBS (in a volume of 10 times of that of starting blood sample) [34]. The white blood cells were collected by 5 min centrifugation at 2000 RPM and lysed in 0.2 ml of ice cold fresh RIPA buffer. After suspension by gentle pipetting, the lysates were set on ice for 15 minutes to increase the total yield of soluble protein. Samples were then sonicated on a 70% setting for 30 sec to shear the DNA, incubated 30–60 minutes on ice, centrifuged on a bench-top microcentrifuge at 12,000×g for 15 minutes at 4°C, and the supernatant collected into a new tube. The protein concentration was determined by the BCA method (Pierce Biotechnology, Rockford, IL).

### Western blot

Two specific antibodies were used to detect ERβ and ERβ2 protein. The H-150 antibody recognizes the N-terminal epitope of rat ERβ (Santa Cruz) and the ERβ2 antibody (gift from Dr. Robert Handa) recognizes the unique 18 aa residues in ERβ2 and does not cross-react with either ERβ, ERα, or other ER isoforms [25]. Thus a protein band on PVDF membrane that is positive for both the H-150 and Handa' antibodies reduced the possibility of a false positive for ERβ2. Antibodies for β-actin, a loading control for cytosol protein, and histone H3, a nuclear protein loading control, were used as internal controls. The relative quantity of ERβ and ERβ2 were normalized by internal controls. The data were collected with a Bio-Rad Molecular Imager ChemiDoc XRS+ system and the optical density was analyzed with the Bio-Rad image lab software.

### Forced Swim Test

Rats were placed in a cylindrical container (40 cm deep, 27 cm in diameter) filled with 30 cm of 30°C water, the 10-min test was videoed, and the amount of time the rats spent swimming or immobile in 10-min was analyzed. Swimming was defined as movement of the forelimbs and hindlimbs without the front paws breaking the surface of the water. Immobility was recorded when there was an absence of any movement other than that necessary to keep the head and nose above the water (when rats were floating in a vertical position) [35], [36]. Before testing, all rats were coded and codes were not broken until the video analyses had been completed.

### Statistics

Statistically analysis was by a Two-way ANOVA followed by a Neuman-Keuls test. In all analyses, differences were considered significant at probability (*p*) values less than 0.05.

## Results

### Removal of ovaries increases the expression of a dominant negative ERβ splice variant, rERβ2

**Figure 2:**
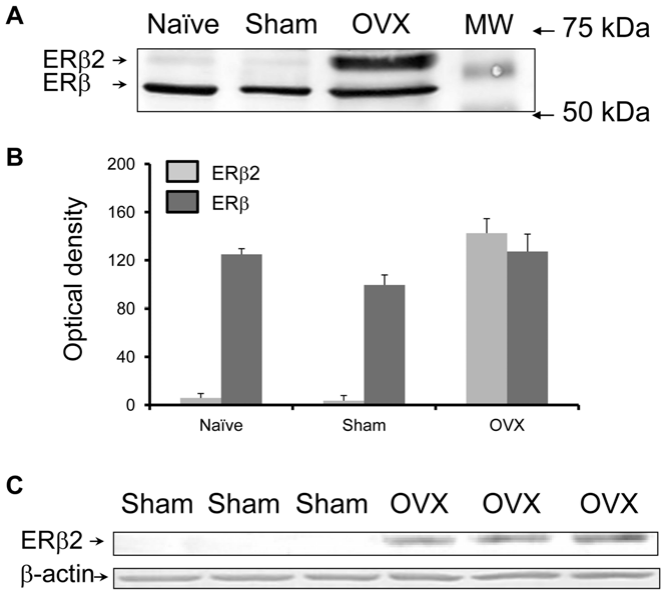
Loss of ovarian hormones increases ERβ2 expression. Each lane was loaded with 30 µg of protein from rat hippocampus (n = 6/group). A. Western blot of ERβ in hippocampus of 6 month old rats that were naive, or sham and OVX for 14 days using h-150 ERβ antibody (Santa Cruz). B. Optical density of the expressed 54 and 57 kDa ERβ immunoreactive proteins using the h-150 ERβ antibody. C. Western blot using the ERβ2 antibody (gift from Dr. Handa) which recognizes the unique 57 kDa 18 amino acid sequence in rERβ2that does not cross-react with either ERβ or ERα.

The role of ovarian hormones on formation of the dominant negative ERβ splice variant, ERβ2 (an intron retention isoform), was first tested in hippocampus of 6 month old reproductive female rats. The ERβ specific antibody (h-150, Santa Cruz) detected two proteins at molecular masses of 54 kDa and 57 kDa (Figure 2A). The 57 kDa larger band is consistent with ERβ2 which has an additional 18 amino acid in the ligand binding domain of ERβ. The ratio of the 57/54 kDa proteins in naïve rats is 4.7%, in sham rats is 3.6%, and in OVX rats is 112% (Figure 2B). To identify the 57 kDa protein band which is recognized by ERβ antibody h-150, an antibody which specifically recognizes the 18 amino acid in ERβ2, but does not cross-react with ERβ or ERα [25] was used. The 57 kDa protein band was only recognized by the antibody against the unique 18 amino acids in ERβ2 (Figure 2C). ERβ2 expression was detectable in 6 month-old female OVX rats 15 days after OVX, but not in sham OVX rats (Figure 2C).

### Early, but not late treatment with exogenous estradiol decreases the OVX-evoked ERβ2 expression in cortex and hippocampus in rats

**Figure 3:**
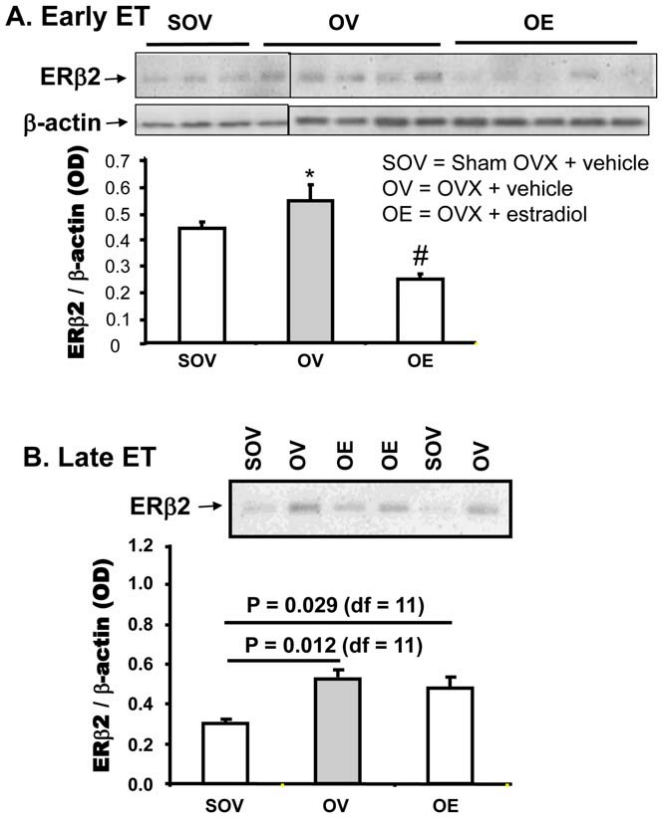
Persistent loss of ovarian hormones diminishes the effects of estradiol on the regulation of ERβ2 expression. ERβ2 protein expression was evaluated by Western blot in hippocampus of rats OVX at 9 months of age in which E2 treatment was started 6 days (A, Early ET) or 180 days (B, Late ET) after OVX. Data is presented as average ± SEM of optical density normalized with optical density of β-actin (n = 8 in SOV, 8 in OV and 9 in OE. * = p<0.05 vs. sham OVX; # = p<0.01 vs. sham OVX.

Figure 3 presents the results of the ERβ2 expression in hippocampus of 9 month old OVX female rats (OV) which were received Early (A) or Late (B) ET (OE) and compared with that in age matched sham OVX rats (SOV). In Early ET group, OVX induced a significant increase (21%, * p<0.05) in ERβ2 immunoreactivity in 9 month old rats within 15 days of OVX compared to sham OVX. Six days of estradiol treatment initiated on day 7 after OVX repressed expression of ERβ2 by 45% compared to OVX 9 month old rats receiving vehicle (#, p<0.01) and which was 25% lower than that in the sham OVX rats.

In Late ET group, ERβ2 expression was consistently higher (47% increase) in hippocampus of OVX rats than the sham OVX rats (Figure 3B). Initiation of estradiol treatment 6 months after OVX had no effect on the repression of ERβ2 which was consistently higher (42%) in hippocampus of Late ET OVX rats compared to sham OVX rats (Figure 3B).

### Duration of ovarian hormone loss and efficacy of estradiol to increase cell proliferation in hippocampus

**Figure 4:**
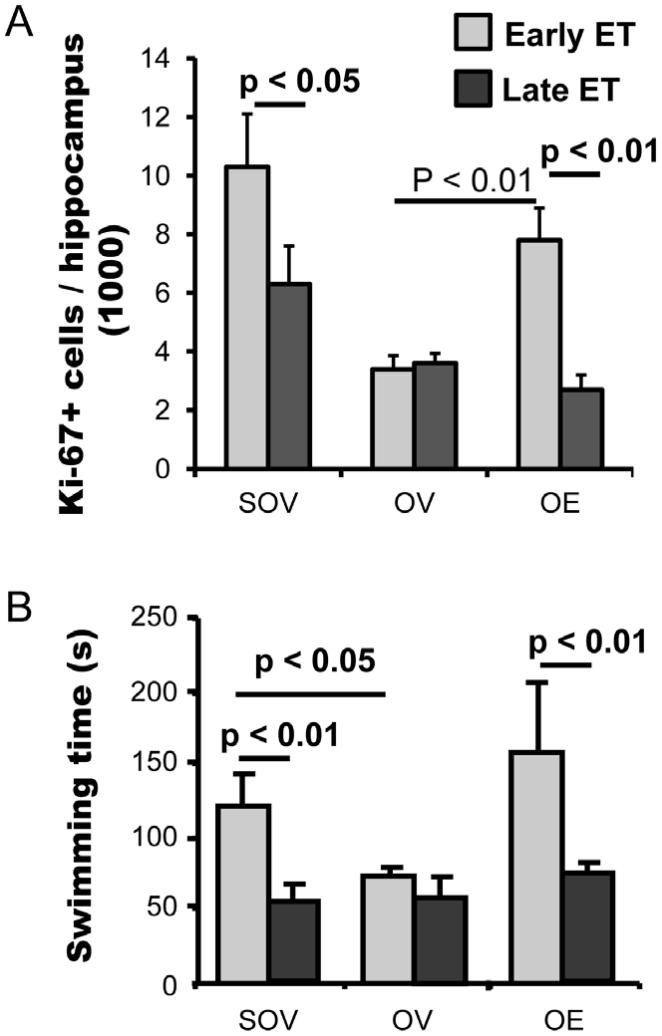
Persistent loss of ovarian hormones diminishes the response to estradiol on hippocampal cell proliferation and duration of forced swim. The effects of estradiol on hippocampal cell proliferation was determined by counting the number of Ki-67 positive nuclei in hippocampus (A); the antidepressant effect of estradiol was evaluated by forced on swim test (B), in rats that received Early or Late ET. Data is presented as average ± SEM (n = 8 in SOV, 8 in OV and 9 in OE).

Ki-67 is an endogenous transient cell proliferation marker expressed in nuclei of actively cycling cells which is not expressed in differentiating quiescent cells. The number of Ki-67-expressing cells was measured using flow cytometry in hippocampus of rats that received Early and Late ET. The Ki-67 positive cells were 41% less (p<0.05) in 15 month old compared to 9.5 month old sham OVX rats (Figure 4A), suggesting an age dependent decrease of hippocampal cell proliferation in female rats. The number of Ki-67 positive cells in 9 and 15 month-old OVX rats were similar despite the different duration of ovary loss, 6 or 180 days, suggesting that ovarian hormones have a major role in hippocampal cell proliferation.

The number of Ki-67 positive cells in the hippocampus of rats receiving Early ET, in which E_2_ treatment was initiated 6 days after OVX, was twice than that of OVX rats not treated with E2 (p<0.01) and not significantly different from that of sham OVX rats (p>0.26). Proliferation of hippocampal cells was not increased in rats receiving Late ET, in which E_2_ treatment was initiated 180 days after OVX (p>0.36), compared to OVX vehicle control. There was a significant difference in hippocampal cell proliferation between the OVX rats that received Early or Late ET (p<0.01). These results suggest that ET increases the proliferation of hippocampal cells, in rats when the estradiol was given soon after OVX, but not after prolonged loss of ovarian hormones.

### Antidepressant efficacy of estrogen therapy correlates with the duration of ovarian hormone loss before therapy is initiated

The forced swim test was performed to evaluate the antidepressant effects of estradiol in Early and Late ET rats (Figure 4B). The swim time of 15 month-old sham OVX rats was 53±15 sec, 57% less than the 9-month old sham-OVX rats (123±27 sec, p<0.01). The swim time of 9 month-old rats OVX for 6 days was 44% less than that of age-matched sham-OVX rats (p<0.05). The swim time of rats receiving estradiol 6 days after OVX was 153±52 sec, significantly higher than that in age matched OVX rats that received vehicle (p<0.01). OVX did not change the swim time of 15 month old female rats and there was no difference in swim time between 9 month-old rats OVX for 6 days and 15 month-old rats OVX for 6 months (OV). The swim time of rats that started to receive estradiol 180 days after OVX was 71±8 sec (Figure 4B), no difference was observed with that in 15 month old sham-OVX and OVX rats. These findings suggest that exogenous estradiol may serve as an anti-depressant agent in rats when the estradiol is given soon after OVX, but not after prolonged loss of ovarian hormones.

### ERβ2 expression in circulating white blood cells (WBC) reflects the expression of ERβ2 in hippocampus

**Figure 5:**
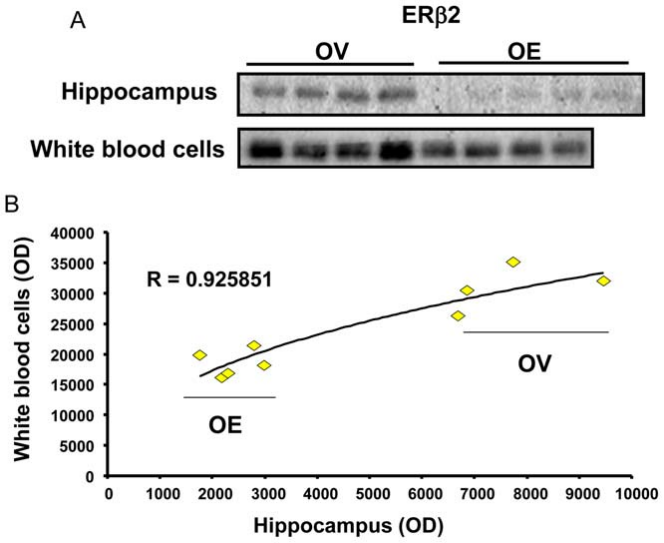
Expression of ERβ2 in white blood cells (WBC) correlates with that in the Hippocampus. The expression of ERβ2 in peripheral blood WBC and hippocampus in rats that received Early ET (OE) or vehicle (OV) was measured by Western blot. A. The representative images of ERβ2 Western blot of hippocampus and WBC. B. Pearson correlation of the optical density of ERβ2 protein expression in extracts of hippocampus and peripheral leukocytes.

If the ERβ2 expression in circulating WBC correlates with that in the hippocampus, then it will be a peripheral, easily accessible, laboratory reference marker for ET prescription in clinic. Therefore, we measured and compared ERβ2 expression protein extraction of the WBC from the whole blood and hippocampus by Western blot. The results of Western blot analysis demonstrated an increase in expression of ERβ2, but not of ERβ, in WBC of the rats OVX for 6 days. Similar to the expression pattern of ERβ2 in hippocampus, Early ET also produced a reduction of expression ERβ2 in WBC of female rats (Figure 5A). The association of the optical densities obtained from the WBC and hippocampus of each samples were analyzed by Pearson Correlation as plotted in Figure 5. The results indicated that the expression levels of ERβ2 in WBC and hippocampus are highly associated (r = 0.93, p<0.05) in OVX rats that received vehicle (OV) or Early ET (OE) (Figure 5B).

## Discussion

The causes for the discrepant results from basic science studies of the effects of estrogen therapy after ovarian hormone loss and the results of the WHIMS trial are no doubt complex. Reanalyzes of the WHIMS data, meta-analyses of early clinical trials, and meta-analysis of studies of ET in bilateral oophorectomy [5], as well as more recent prospective studies, support the theory of a critical period or therapeutic window soon after menopause during which time the initiation of estrogen therapy maintains normal neural and cognitive functions, while initiation of ET well after menopause is ineffective or harmful.

The molecular mechanisms for this window are unknown. Recent studies suggest that the C terminus of Hsc70-interacting protein (CHIP)-mediated degradation of hippocampal estrogen receptor-α [37] may serve as a molecular mechanism for the critical therapeutic window for post-menopausal ET [37], [38]. We present another potential mechanism, the increased expression of the dominant negative ERβ isoform, ERβ2, which appears to be a crucial determinant of the neuroprotective and neurogenic efficacy of ET responsible, at least in part, for the loss of estrogen response after protracted absence of ovarian hormones in a rat model of menopause.

The 18-amino acid insertion of ERβ2 lies within helix 6, a structure immediately following the β-turn within the ligand binding domain and in the center of the ligand binding pocket [39]. Recombinant protein generated by *in vitro* transcription/translation showed that ERβ2 had markedly reduced ligand binding (K(D) = 17.7+/−4.7 nM) compared with ERβ bound ^3^H-estradiol (K(D) = 0.56+/−0.19 nM) [40], demonstrating that the 18 amino acids insertion significantly alters the structure of the ERβ ligand binding pocket and impedes binding to ligands. Because their DNA binding domains are the same, ERβ2 and ERβ have similar binding affinity for palindromic estrogen responsive elements (EREs) *in vitro* and *in vivo* [40]. ERβ2 has little transcriptional activity except at high ligand concentrations in NIH 3T3 cells with transactivated ERE-tk-CAT reporter genes, due to low ligand binding affinity. Co-transfected ERβ2 has been found to significantly inhibit the transcriptional activities of ERα and ERβ in several studies [22], [25], [40]–[42]. The most plausible mechanism is that ERβ2 competes as a homodimer or heterodimer with the homodimers of ERα or ERβ in binding to ERE. Both human and rodent ERβ2 forms heterodimers of ERα/ERβ2 or ERβ/ERβ2, as demonstrated by gel shift assay [17], [22], [41], and the ERα/ERβ2 heterodimer shows significantly higher affinity to ERE than that of the homodimer of ERα [42].

Nuclear receptors form strong dimers that are essential for their function as transcription factors and ligand binding affects dimer stability. ER dimerization is ligand independent [43], [44]. Unliganded ER-LBDs exist as stable dimers with a slow dissociation rate (t1/2 = 39±3 min at 28 C) which is further slowed (≤7-fold) by the addition of various ligands [45]. Dimerization of ERα and ERβ is E_2_- and antiestrogen (EM-652)-independent in human embryo kidney 293 cells using the fluorescence resonance energy transfer (FRET) technique [46] and bioluminescence resonance energy transfer (BRET) assays [44]. The dimers of ERβ2/ERβ2, ERβ2/ERβ or ERβ2/ERα are predicted to bind to E2 at least 30 times less [25], [41], [42], thus their transcriptional activity is similarly reduced. Hence the efficacy of estradiol is diminished when expression of ERβ2 increases, as occurs after loss of ovarian estrogen. While the hypothesis is that in the absence of E_2_ in the rat and human the estrogen insensitive ERβ2 accumulates and obstructs normal function of the remaining ER [22], [41], [42], the possibility of the accumulation of another ligand for ERβ2 that causes it to act as a repressor of normal ER function should not be discounted [42].

The relevance of our rat model to the human after menopause is based on the following. First, the human ERβ2 and rat ERβ2 are functionally comparable. Both hERβ2 and rERβ2 differ from ERβ in the ligand-binding domain, both exhibit similar dominant negative function, and both form dimers with ERα or ERβ [18], [25], [26], [47]–[50]. Second, estropause in rodent shares similarities with human menopause. The rodent estrous cycle is 4–5 days long, consisting of proestrus, estrus, diestrus I, and diestrus II. Rats begin to show irregular cycles at 9–12 months, defined by prolonged cycles, interspersed with the normal 4–5 days rhythm, followed by a persistent acyclic, anestrus state with no ovulation between 15 and 23 months of age [28], [29]. Rat estropause differs from monkey and human menopause in that aged rats retain a much larger number of primary oocytes, resulting in higher estrogen levels at the onset of persistent diestrus than those seen in primates after menopause [28] [51], [52]. To limit this difference, the ovaries were removed from rats entering estropause, thus providing a model that mimics both the age and ovarian hormone status of the post menopausal woman. The female rats receiving estradiol on day 7, or 181 after OVX at 9 months of age is analogous to estrogen therapy in a human early and decades later after menopause, respectively. The ages of the rats used, 6, 9 and 15 months, are thought to correspond with adult reproductive, peri-menopasue, and late post-menopause in women.

In the current study ERβ2 expression increased in the brains of intact rats with age and estropause, natural ovarian failure, as well as with ovariectomy. The progressive increase in ERβ2 from 6, to 9 to 15 months of age in the sham control correlated with a significant decrease in NPC proliferation in the hippocampus, as well as swim time in a forced swim test. Similarly, Nerve growth factor and brain Insulin-like Growth Factor decrease in with age rats [53]–[56].

Loss of neurons and a decrease in NPC proliferation are associated with depression and dementia, and preservation of NPC proliferation is suggested as a mechanism for the antidepressant effect of estrogen treatment after menopause and ovariectomy [57]–[60]. Others and we have demonstrated a time dependent loss of the effect of estradiol on NPC proliferation in dentate gyrus SGZ in OVX rats [30], [61]. Unbiased stereology in rats at 6 months of age demonstrated that compared to the vehicle treated OVX group, estradiol administration initiated 6 days after OVX increased SGZ NPC proliferation by 40%, while ET initiated 28 days post-OVX had no significant effect on OVX-induced decline of NPC proliferation [30], [61]. Using a similar protocol, we used unbiased steriology to measure BrdU positive cells in hippocampal SGZ in rats in which ET was initiated 14 days after OVX and observed a 13.2% increase in proliferation (unpublished data).

Ki-67 is an endogenous transient cell proliferating marker expressed in nuclei of actively cycling and does not express in the differentiating quiescent cells. In our previous study we observed a 43% increase in hippocampal cell proliferation upon estradiol treatment of OVX rats by counting Ki-67 positive cell numbers using flow cytometry [32]. While analysis by flow cytometry does not determine the type of proliferating cell, this relative increase is similar to the 40% increase of BrdU cells in SGZ in the estradiol treated group reported in Tanapat's study [30], suggesting that the cell proliferation detected by Ki-67 positive cells in whole hippocampus reflects the dividing potential of NPC in SGZ, though proliferation of other cells may also be involved.

In the current study hippocampal cell proliferation and swim time were not significantly different between intact 15-month-old rats and those ovariectomized 6 months before at 9 months of age, when ovarian function in rats normally declines. This suggests that this model is appropriate for comparison to the WHIMS study in which ET was initiated a decade or more after natural menopause in many of the women. ET initiated 6 days after OVX in rats reversed the decrease in hippocampal cell proliferation produced by OVX, however ET initiated 180 days after OVX, a situation analogous to initiating ET a decade or more after natural menopause, did not.

In summary, our study confirmed and extended the evidence for a consistent progressive increase in ERβ2 associated with the loss of ovarian hormones. The accumulation of the dominant negative ERβ2 correlated with duration of ovarian hormone loss and the inability of ET to reverse the decrease in neuronal proliferation and function and performance in the forced swim test for depression in rats. Accelerated ERβ2 expression and accumulation in women may be a mechanism for the loss of efficacy of estrogen therapy if treatment is delayed for too long after menopause. Significant risks of ET in women have been identified, particularly in those in which ET is initiated long after menopause. Expression of ERβ2 in circulating leukocytes paralleled that in the brain, suggesting the potential for using WBC ERβ2 levels as a clinically useful biomarker to identify the therapeutic window for effective ET. Measurement of ERβ2 expression in peripheral WBC could provide a guide for when to initiate post-menopausal estrogen therapy and to avoid therapy when the risk/benefit ratio is unfavorable. Furthermore, determination of the factors leading to increased ERβ2 expression could lead to targeted non-estrogen therapies and/or expand the therapeutic window and delay the depression and cognitive loss that has been associated with menopause.
